# Dietary Tart Cherry and Fructooligosaccharides Promote Bone Health via the Gut Microbiota and Increased Bone Formation

**DOI:** 10.3390/nu17172829

**Published:** 2025-08-30

**Authors:** Pelumi Adedigba, John A. Ice, Sanmi E. Alake, Bethany Hatter, Proapa Islam, Ashlee N. Ford Versypt, Trina A. Knotts, Jerry Ritchey, Edralin A. Lucas, Brenda J. Smith

**Affiliations:** 1 Indiana Center for Musculoskeletal Health, Indiana School of Medicine, Indianapolis, IN 46202, USA; padedigb@iu.edu; 2 Nutritional Sciences Department, Oklahoma State University, Stillwater, OK 74078, USA; john.ice@va.gov (J.A.I.); sanmi.alake@okstate.edu (S.E.A.); bhatter@ostatemail.okstate.edu (B.H.); proapa.islam@okstate.edu (P.I.); edralin.a.lucas@okstate.edu (E.A.L.); 3 Department of Chemical and Biological Engineering, University at Buffalo, Buffalo, NY 14260, USA; ashleefv@buffalo.edu; 4 Department of Surgery, Center for Alimentary and Metabolic Science, UC Davis Health, Sacramento, CA 95817, USA; taknotts@ucdavis.edu; 5 Veterinary Pathobiology Department, Oklahoma State University, Stillwater, OK 74078, USA; jerry.ritchey@okstate.edu; 6 Department of Obstetrics and Gynecology, Indiana School of Medicine, Indianapolis, IN 46202, USA

**Keywords:** gut-bone axis, prebiotics, microbiota, antibiotics, short chain fatty acids, fructooligosaccharides, tart cherry, osteocytes, histomorphometry

## Abstract

**Background/Objectives:** Fructooligosaccharides (FOS) and dried tart cherry (TC) are examples of simple and complex (i.e., within a food matrix) prebiotics that have demonstrated promising osteoprotective activity. In this study, we examined how dietary supplementation with TC or FOS shapes the gut-bone axis to promote bone accrual in young adult mice, and the role of the gut microbiota in mediating these responses. **Methods:** Studies were performed using 10-wk-old female C57BL/6 mice (n = 10–12/group) fed a control diet or control diet supplemented with 10% TC or FOS for 10 wks alone or in combination with an antibiotic/anti-fungal cocktail to suppress the gut microbiota. The bone phenotype was characterized by dual-energy X-ray absorptiometry, micro-computed tomography and static and dynamic bone histomorphometry. The gut-microbiota was profiled and short chain fatty acids (SCFA) were assessed based on 16S rRNA profiling and gas chromatographic techniques, respectively. **Results:** FOS and TC enhanced bone structure, with FOS yielding more pronounced benefits across cortical and trabecular compartments. These skeletal improvements with FOS occurred in the absence of systemic changes in bone turnover markers but were accompanied by increases in local bone formation, osteoblast and osteocyte numbers, and bone mineralization in the femur. Both diets altered gut microbiota composition and increased fecal concentrations of the most abundant SCFAs (i.e., acetate, propionate and butyrate), but the response was greater with FOS. Suppression of the gut microbiota and fecal SCFAs with the antibiotic/anti-fungal cocktail inhibited the effects of FOS and TC on cortical bone, but induced unexpected improvements in the trabecular bone. **Conclusions:** These findings demonstrate differential effects of simple and complex prebiotics on the gut-bone axis in young adult female mice and support a role for SCFA in the cortical bone response, but not in the trabecular bone response with this model of gut microbiota suppression.

## 1. Introduction

The gut microbiota plays a crucial role in maintaining bone tissue by influencing key processes, including bone formation and resorption [1,2]. These effects can be mediated through systemic factors, including gut-derived metabolites, hormones, and cytokines, as well as and local signaling pathways (e.g., Wnt signaling and osteoclastogenesis) that regulate bone homeostasis [3,4,5]. In the absence or ablation of the gut microbiota as in the case of germ-free (GF) mice or mice on antibiotic (ABX) treatment, bone mass, biomechanical properties, and microarchitecture are altered [1,2,6]. Drugs, such as glucocorticoids that are used in the treatment of chronic inflammatory conditions (e.g., inflammatory bowel disease, rheumatoid arthritis, and chronic obstructive pulmonary disease) and the bone anabolic agent intermittent parathyroid hormone (iPTH), have been reported to mediate their effects on the bone through the gut microbiota [7,8]. For instance, chronic glucocorticoid use induces bone loss and imbalances in intestinal microbial communities, which leads to a compromise in the gut barrier. Administration of probiotics and prebiotics restored the gut barrier and prevented bone loss [9,10]. Additionally, postmenopausal women with osteoporosis have reduced microbiota diversity and increased intestinal permeability compared to age-matched controls without osteoporosis, suggesting a link between gut dysbiosis and postmenopausal osteoporosis development [11,12]. Mechanical loading, which has been shown to promote bone formation and increase bone mass, fails to achieve these effects in mice depleted of their gut microbiota [13]. Combined, this growing body of pre-clinical and clinical evidence highlights the role of the gut microbiota as a key regulator of skeletal health and a promising target for dietary interventions.

Prebiotics are non-digestible food components that selectively stimulate the growth and activity of beneficial gut microbiota, by serving as substrates that can modulate immune responses, improve gut barrier integrity, and improve some aspects of the host’s health [5,14]. Prebiotics can be classified based on their composition as either simple or complex. Simple prebiotics include a single class of compounds such as fructooligosaccharides (FOS), galactooligosaccharides (GOS), resistant starch, polyphenols, or other phytochemicals. In contrast, complex prebiotic sources are typically prebiotics that exists within a food matrix (e.g., chicory, tart cherries, plums, onions and garlic), which can influence where and how rapidly they are metabolized [15,16].

The simple prebiotic, FOS, composed of linear fructose oligomers has been studied for its benefits on bone health [17,18]. In ovarian hormone deficient rats, FOS enhanced intestinal calcium and magnesium absorption, increased bone mineral content, and suppressed bone resorption [19,20,21]. Clinical studies have linked FOS intake to higher bone mineral density (BMD) in adolescents and reduced bone turnover markers in postmenopausal women [22,23]. Recently, we reported that a short chain FOS that is enzymatically synthesized from sucrose, enhances bone accrual in young adult female mice in conjunction with increasing osteocyte density and regulators of osteoblast differentiation and activity in young adult mice [24]. The beneficial effects of FOS on the gut-bone axis have been attributed primarily to alterations in the gut microbiota composition and the subsequent fermentation of non-digestible carbohydrates to produce short-chain fatty acids (SCFAs) [24,25].

Tart cherries (*Prunus cerasus*; TC) are regarded as a fruit with potential prebiotic activity that improves bone health, through its fermentable fibers and polyphenols that support gut microbiota metabolism [26,27]. TC is widely recognized for its health-promoting properties, primarily attributed to its anti-inflammatory and antioxidant effects [28,29]. In addition to providing some FOS (0.33 g/100 g), TC contains a diverse profile of polyphenolic compounds, including anthocyanins and hydroxycinnamic acids, that have prebiotic activity, influencing gut microbiota composition and function [26,30,31,32]. Preclinical evidence suggests that these compounds can enhance the production of gut-derived metabolites, including SCFAs, which support intestinal and systemic health [33,34]. TC have been investigated in several clinical trials, with studies reporting potential benefits on vascular function in men, muscle recovery in male and female runners, sleep quality, and the management of osteoarthritis [29,35,36,37,38,39]. We reported that consuming tart cherry juice for 90 d reduced serum tartrate resistant acid phosphatase 5b (TRAP5b), a bone resorption marker, in postmenopausal women [40]. In preclinical studies, our lab and others have shown that TC supplementation protects against age-related trabecular bone loss and the deterioration of bone associated with inflammation in a mouse model of rheumatoid arthritis [27,40,41].

FOS and TC represent examples of simple and complex prebiotics that have each been shown to exert beneficial effects on bone in both pre-clinical and clinical studies. Despite their effects on bone, a comparison of their influence on the gut microbiota and the mechanisms through which their effects on bone are mediated have not been investigated. Thus, it is important to investigate how dietary supplementation of these two very different prebiotics affects bone structural properties and remodeling and the role of the microbiota in these responses.

## 2. Materials and Methods

### 2.1. Animal Care and Experimental Design

All procedures adhered to the guidelines set forth by the Oklahoma State University (OSU) Institutional Care and Animal Use Committee. Eight-week-old female C57BL/6 mice (n = 96; Taconic Biosciences, Germantown, NY, USA) were acclimated for 2 weeks and fed one of three diets: control diet (CON; AIN-93M), tart cherry (TC; 10% *w*/*w*), or short chain FOS (10% *w*/*w*) for 10 wks. Pitted flash-frozen Montmorency tart cherries were purchased (Shoreline Fruits at Peterson Farms, Shelby, MI, USA), lyophilized, and ground into powder. FOS (NUTRAFLORA^®^, FB P-95, Ingredion Inc., Westchester, IL, USA) was purchased in powder form. Diets were formulated based on the semi-purified AIN-93M diet and adjusted to have similar macronutrient, calcium, and phosphorous content [42]. A separate set of mice had antibiotics (ABX) added to their drinking water to further investigate the role of the gut microbiota in the response to FOS and TC. The antibiotic cocktail included metronidazole (1 mg/mL; Cayman Chemical, Ann Arbor, MI, USA 9002409), ampicillin (1 mg/mL; Cayman Chemical 14417), vancomycin (0.5 mg/mL; VWR V0990), and neomycin (1 mg/mL; N6386 Sigma Aldrich, St. Louis, MO, USA), incorporated into the drinking water. This cocktail has been shown to suppress almost all bacteria population within the gut [43,44]. An anti-fungal, amphotericin B (0.01 mg/mL; Sigma Aldrich A9528), was also added to the cocktail to reduce the risk of overgrowth of fungi with the suppression of bacterial communities within the gastrointestinal tract as has been reported [45]. Mice were group housed (four mice/cage) using a wire grid on top of the bedding in shoe-box cages to avoid coprophagy and had access to deionized water ad libitum. Food intake was recorded daily, and body weights were recorded on a weekly basis. Animals were housed in the OSU Laboratory Animal Research Facility with controlled access and environmental conditions (12-h light and dark cycle).

At the end of the study, fecal samples were collected and stored at −80 °C for SCFA analysis. Mice were fasted for 3 h and anesthetized with ketamine/xylazine cocktail (100 mg/10 mg per kg bodyweight). Whole body dual-energy X-ray absorptiometry (DXA) scans (LunarPIXI, GE Medical Systems, Madison, WI, USA) were performed, and mice were exsanguinated via the carotid artery. Blood was processed for serum prior to storage at −80 °C for future analysis. Cecal contents were flushed with ice cold, sterile PBS, centrifuged and the supinate decanted prior to weighing and storage at −80 °C for microbiota profiling. One femur specimen was cleaned of soft tissues, and the bone marrow was flushed with ice cold Dulbecco’s Modified Eagle Medium (DMEM) media and processed for fluorescence activated cell sorting (FACS) analysis. The second femur and the 5th lumbar vertebra were collected, cleaned of soft tissue, and either stored in 70% ethanol or fixed in 10% neutral buffered formalin (NBF) and stored for micro-computed tomography (µCT) (µCT40, SCANCO Medical, Wangen Bruttisellen, Switzerland). Following flushing of the intestine with ice cold PBS, the ileum was collected, Peyer’s patches were removed, and the remaining ileum was processed for FACS analysis. The colon length was measured, and the lamina propria was collected and stored in RNAlater for gene expression analysis.

### 2.2. Whole Body Composition and Bone Densitometry Assessment

Whole body DXA scans were analyzed to assess body composition (i.e., lean mass, fat mass, and body fat percentage), bone mineral area (BMA), content (BMC), and density (BMD) with Series Software Version 1.4x (GE Lunar PixiMus, Madison, WI, USA).

### 2.3. Micro-Computed Tomography (µCT) Analyses

Quantification of trabecular and cortical bone microarchitecture within the femur and the fifth lumbar vertebra was performed using X-ray µCT. The distal femoral metaphysis and mid-diaphysis were used to analyze trabecular and cortical bones, respectively. Scans of the femoral distal metaphysis were performed at a resolution of 2048 × 2048 pixels and were analyzed by evaluating approximately 140 slices (0.840 mm) within the volume of interest (VOI). Analysis of the lumbar vertebra was performed by acquiring images at a resolution of 1024 × 1024 pixels and a VOI of approximately 170 slices (2.7 mm) medial to the dorsal and caudal growth plates. Only the secondary spongiosa was considered for trabecular bone analysis. Trabecular bone parameters included bone volume per total volume (BV/TV), trabecular number (TbN), trabecular thickness (TbTh), trabecular space (TbSp), connectivity density (ConnDens), and structural model index (SMI). The cortical bone of the femur was evaluated by analyzing approximately 30 slices (180 μm) within the VOI at mid-diaphysis. Cortical bone parameters accessed included cortical porosity, thickness, area, and the medullary cavity area. All µCT analyses utilized a threshold of 350, with a sigma of 1.2 and a support of 2.0.

### 2.4. Serum Bone Biomarkers

Systemic biomarkers were assessed in serum samples collected at the end of the study using commercially available ELISA kits. These included biomarkers of osteoblast activity (P1NP), osteoclast activity (CTX-I) (Immunodiagnostic Systems Inc., Fountain Hills, AZ, USA), insulin-like growth factor-1 (IGF-1) (R&D Systems, Minneapolis, MN, USA), and IGF binding protein-3 (IGFBP-3) (Sigma-Aldrich).

### 2.5. Gut Microbiota Analyses

Total DNA isolation was carried out on frozen cecal samples using the QIAamp PowerFecal DNA kit (Qiagen, Hilden, Germany) according to manufacturer’s instructions. Sample libraries were prepared and analyzed by barcoded amplicon sequencing at the Mouse Metabolic Phenotyping Center, University of California, Davis (Davis, CA, USA). The DNA samples were purified and the V4 region of the 16s rRNA amplified using PCR with the following primers: F341 (5′-CCTACGGGNGGCWGCAG-3′) and R806 (5′-GGACTACNVGGGTWTCTAAT-3′). High-throughput sequencing was performed with Ilumina MiSeq paired end 250-bp run. Data derived from sequencing was processed using QIIME2 for 16S based microbiota analyses. Demultiplexed paired end sequences with barcodes and adapters removed were analyzed using Qiime 2 version 2023.5.1. DADA2 package version 1.26.0 was used for quality filtering and generating amplicon sequence variants. Taxonomic classification of OTUs was done with a pre-trained Naïve Bayes taxonomy classifier called the Silva_138_99% OTUs. Diversity analyses were run on the resulting OTU/feature.biom tables to provide both phylogenetic and non-phylogenetic metrics of alpha and beta diversity. Tables of taxonomic counts and percentage (relative frequency) were generated.

### 2.6. Fecal SCFA Analyses

Fecal SCFAs were assessed using gas chromatography techniques as previously described [46]. Briefly, samples were lyophilized and granulated into a powder, and 150 mg was used in the assay. Next, 250 µL of 7.14 M sulfuric acid, 45 µL internal standard (1 mM 2-ethyl butyric acid in 12% formic acid) was added to the tube, followed by two rounds of extraction with 1 mL diethyl ether. Aliquots of approximately 500 µL of the organic phase were collected into a GC sample vial and transferred to an Agilent 6890N GC system with flame ionization detector and automatic liquid sampler (Agilent Technologies, Santa Clara, CA, USA) for analysis. A 5-point calibration was performed using standard solutions of acetic, propionic, butyric, and valeric acids (Sigma-Aldrich).

### 2.7. Intestinal Bacterial Load

To determine whether the antibiotic cocktail suppressed the gut microbiota within each diet group, a bacterial load assay was performed on fecal and cecal samples [47]. In short, genomic DNA was extracted from fecal and cecal contents using the QIAamp PowerFecal DNA Kit (QIAGEN), following the manufacturer’s protocol. The method used to evaluate DNA quantity was spectrophotometric analysis; DNA concentration and purity were measured using a NanoDrop spectrophotometer (Thermo Fisher Scientific, Waltham, MA, USA).

### 2.8. Dynamic and Static Bone Histomorphometry

For histomorphometric analysis, fluorochrome labeling of the bones was performed by subcutaneous injections of calcein and alizarin red, administered 7 and 2 days before sacrifice. A subset of femurs from each group were dissected, fixed, and embedded in methyl methacrylate. Static histomorphometric analysis of non-decalcified bone sections was performed in the distal femur metaphysis. Von Kossa stain was performed and osteocytes and osteoblasts quantified. Silver nitrate stain was used for visualizing osteocyte lacuna and the canalicular system. Femur samples were processed at the Indiana Center for Musculoskeletal Health Histology and Histomorphometry Core. Histomorphometric analysis was performed using an OsteoMeasure high resolution digital video system (OsteoMetrics Inc., Decatur, GA, USA) applying units recommended by the Histomorphometry Nomenclature Committee of the American Society for Bone and Mineral Research [48].

### 2.9. Flow Cytometry

Bone marrow was flushed using incomplete DMEM media. Red blood cells (RBCs) and platelets were subjected to lysis in a dark environment at room temperature for a duration of 5 min, using lysing buffer (BD Bioscience, Franklin Lakes, NJ, USA). To halt the RBC lysis process, incomplete DMEM was added, followed by centrifugation. The resulting cell pellet was re-suspended in 2 mL of complete media composed of DMEM media, 0.5% BSA, and 10 mM EDTA, pH adjusted to 7.4.

The ileum was flushed with RPMI medium, 2% FBS, and 1 mM dithiothreitol (DTT), and the Peyer’s patches and adhering fat tissues were removed. Excised ileal sections were incubated with HBSS (without phenol red) + 5 mM EDTA (pH~7.4) at room temperature to remove epithelial cells, followed by 0.20 mg/mL collagenase type VII digestion (Sigma-Aldrich), a process that was repeated ×3. Cells were collected, resuspended, and filtered through a sterile 70 µm filter and subjected to separation on gradients containing 40% Percoll (20 mL 100% Percoll, 30 mL HBSS with phenol red) and 80% Percoll (40 mL 100% Percoll, 10 mL HBSS). Lymphocytes located at the interface of these gradients were collected and subsequently washed three times with complete media.

Viable cells from the bone marrow and ileum (2 × 10^6^/sample) were transferred into 12 × 70 mm round bottom FACS tube and incubated with live/dead stain for 30 min. Next, the cells underwent staining with surface markers (CD3, CD4, CD8, and CD25). Subsequently, cells were fixed using the mouse FOXP3 fixation/permeabilization buffer (BD Biosciences) for 30 min and protected from light. Cells were then stained for FOXP3, RORγt, and IL-17. Antibodies used included CD3e–eFluor 450 and FOXP3–eFluor 660 (eBioscience, San Diego, CA, USA), CD4–PerCP-Cy5.5 and CD25–PE-Cy7 (BD Biosciences), and CD8–Alexa Fluor 488 and IL-17A–PE (BioLegend, San Diego, CA, USA). Flow cytometry was performed using a BD FACSAria III (BD Biosciences) at the Core Flow Cytometry Laboratory, Center for Veterinary Health Sciences, Oklahoma State University. Data were analyzed with FlowJo (v10.8), using fluorescence minus one (FMO) and unstained controls for gating.

### 2.10. Statistical Analysis

All data, with the exception of the microbiota data, were analyzed using SAS software (Version 9.43). Prior to analyses, outliers defined as values greater than ±2 SD from the mean were excluded from analyses. Assumptions of normality and equal variance were evaluated using the Shapiro-Wilks and Levene’s tests, respectively. Data not meeting normality assumptions were log-transformed; if normality assumptions were still unmet, Friedman’s test was applied.

For cecal microbiota analysis, alpha and beta diversity metrics were calculated using the QIIME pipeline (QIIME 2). Rarefaction was performed to standardize sequencing depth across samples, with a sampling depth set at 23,580 sequences per sample. Alpha diversity was assessed using the Shannon index, while beta diversity was evaluated using the Jaccard index followed by PERMANOVA pairwise comparisons. Differences in taxonomic abundance at multiple levels (Phylum, Class, Order, Family, Genus, and Species) were analyzed using the Analysis of Composition of Microbiomes with Bias Correction (ANCOM-BC). To control multiple comparisons, adjusted *p*-values were calculated using the Benjamini–Hochberg (BH) method.

The effects of dietary treatments (CON, TC, or FOS) were assessed using one-way ANOVA, and post hoc comparison was conducted using Tukey’s test. *t*-tests were performed on outcomes of interest to understand the influence of the ABX cocktail on bone phenotype of mice consuming the control diet (CON vs. CONABX). Next, we assessed the role of gut microbiota in diet-induced changes in body composition and the bone phenotype, using two-way ANOVA with Diet and ABX as factors. Where statistically significant Diet × ABX interactions were observed, post hoc comparisons were conducted using Tukey’s test (*p* < 0.05). Finally, because of the exploratory nature of the dynamic and static bone histomorphometry analysis utilizing n = 4 mice/group, *t*-tests comparing either FOS or TC to the CON were performed. All data are presented as mean ± standard error, with α set at 0.05.

## 3. Results

### 3.1. FOS and TC Alter Body Composition and Bone Phenotype

**Figure 1:**
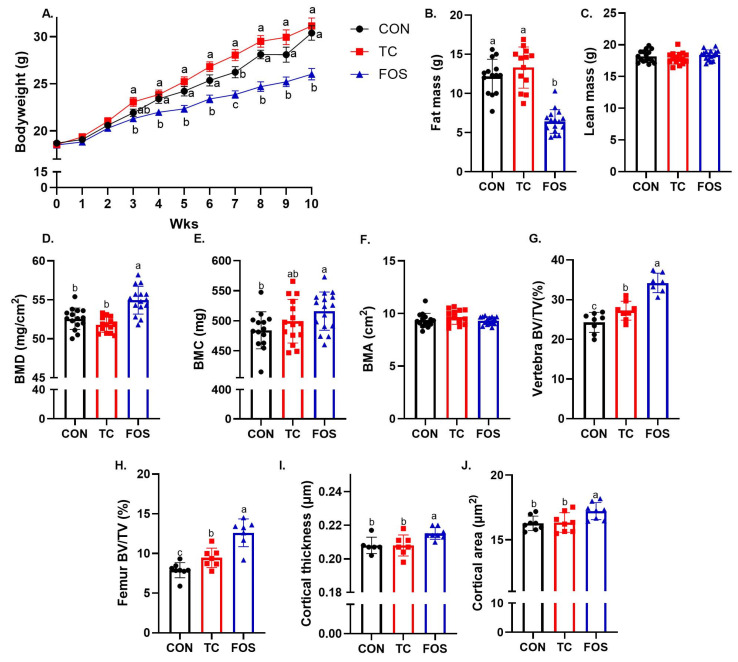
Effect of dietary supplementation with Control (CON), tart cherry (TC) or fructooligosaccharides (FOS) for 10 wks on: (**A**) body weight over time, whole body (**B**) fat mass, (**C**) lean mass, (**D**) bone mineral density (BMD), (**E**) bone mineral content (BMC), (**F**) bone mineral area (BMA), and (**G**) lumbar vertebra trabecular bone volume relative to total volume (BV/TV), (**H**) distal femoral trabecular BV/TV, (**I**) femoral midshaft cortical thickness and (**J**) cortical area. Data presented as mean ± standard error (SE). For body weight (**A**), means that do not share the same superscript letter at a given timepoint are statistically different from each other. In all other figures, bars not sharing the same superscript letter differ significantly (*p* < 0.05, one-way ANOVA with Tukey’s post hoc test).

All diet groups gained weight throughout the 10-wk study. However, by the third week the FOS treated group had lower bodyweight (*p* < 0.05) compared to mice on the TC and CON diets (Figure 1A). Slower weight gain in this group persisted throughout the remainder of the study. The lower body weight exhibited in the FOS-treated mice coincided with less whole body fat mass (*p* < 0.0001) (Figure 1B) and a lower percentage of body fat (*p* < 0.0001) (Supplementary Table S1) compared to the TC and CON groups at the end of the study. Interestingly, there was no difference in lean mass between groups, indicating that the lower bodyweight in the FOS group was due to alterations in body fat only and not lean mass (Figure 1C).

At the end of the study, whole body DXA scans revealed that the FOS group had a higher whole-body BMD (*p* < 0.0001) compared to the TC and CON diet groups (Figure 1D). This was due to mice consuming the FOS diet having higher whole-body BMC (*p* < 0.0001) compared to CON, but there was no difference in BMC between the FOS and TC groups (Figure 1E). There were also no observable effects of dietary treatment on whole body BMA (Figure 1F). 

To further understand the effects of the dietary treatments on bone microarchitecture, µCT imaging was used to assess alterations in the trabecular bone within the axial skeleton (i.e., lumbar vertebrae), and the trabecular and cortical bone structural characteristics in appendicular skeleton (i.e., femur) following 10 wks of treatment. In the lumbar vertebrae, FOS and TC dietary treatments improved trabecular BV/TV (*p* < 0.05) compared to CON, but the magnitude of the response was greater with the FOS treatment compared to the TC (Figure 1G). The FOS group had higher TbTh (*p* < 0.0001) than the TC and CON groups, but there were no effects of dietary treatment on TbN and TbSp (Supplementary Table S1). Trabecular connectivity density within the vertebral body was higher (*p* < 0.05) in FOS and TC groups compared to CON mice. Mice on the FOS- and TC-supplemented diets also had lower trabecular bone SMI (*p* < 0.0001) compared to CON (Supplementary Table S1), which is consistent with a more plate-like and potentially biomechanically stronger structure. In the distal femur metaphysis, the FOS and TC treated groups also exhibited an improved trabecular BV/TV (Figure 1H). The FOS and TC groups had higher TbN (*p* < 0.05) compared to the CON group, with the changes being more pronounced in the FOS group (Supplementary Table S1). While there were no differences in TbTh between the CON and FOS groups, TC exhibited lower TbTh (*p* < 0.01) than the other dietary groups. TbSp was lower in the FOS and TC groups (*p* < 0.0001) compared to CON. Trabecular connectivity density within the femur was higher (*p* < 0.01) in FOS and TC treated mice compared to CON. The FOS group also had a lower SMI (*p* < 0.05) compared to CON (Supplementary Table S1).

Cortical bone was evaluated at the femur mid-diaphysis. Only mice fed the FOS diet had higher cortical thickness and area (*p* < 0.05) compared to mice on the CON diet (Figure 1I,J). No differences were observed between CON and FOS or CON and TC groups in cortical porosity, but mice consuming the FOS diet had lower porosity (*p* < 0.05) compared to mice consuming the TC diet (Supplementary Table S1).

### 3.2. FOS and TC Effects on IGF-1 and Bone Biomarkers

**Figure 2:**
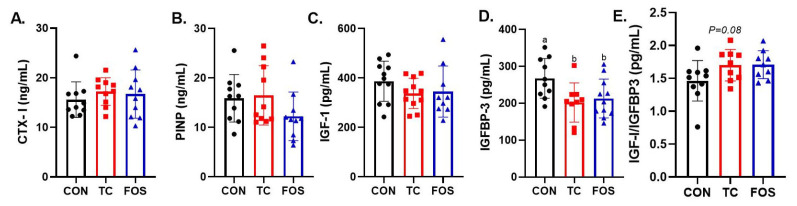
Dietary tart cherry (TC) and fructooligosaccharides (FOS) alter systemic biomarkers of bone formation and IGF axis regulation: (**A**) *C*-terminal telopeptide of type I collagen (CTX-I), (**B**) procollagen type I N-terminal propeptide (P1NP), (**C**) insulin-like growth factor 1 (IGF-1), (**D**) IGF-binding protein 3 (IGFBP-3), and (**E**) IGF-1/IGFBP-3 ratio. Data are presented as mean ± standard error (SE). Bars not sharing the same superscript letter differ significantly (*p* < 0.05, one-way ANOVA with post hoc test).

After 10 wks of dietary treatment, there were no alterations in serum bone resorption or formation markers, CTX-I (Figure 2A) and P1NP (Figure 2B) in response to FOS and TC supplementation. Due to reports that IGF-1 is involved in the gut microbiota’s influence on bone [2], serum IGF-1 and its primary binding protein (i.e., IGFBP-3) were assessed. No effects of dietary treatments on serum IGF-1 were noted (Figure 2C), but the TC and FOS groups exhibited lower serum IGFBP-3 (*p* < 0.05) compared to the CON group (Figure 2D). As a result, the ratio of IGF-1 to IGFBP3 tended to be increased (*p* = 0.08) in the TC and FOS treated groups compared to CON, but this response did not reach statistical significance after 10 wks (Figure 2E).

### 3.3. FOS and TC Shift Gut Microbiota Composition and Increase SCFAs

**Figure 3:**
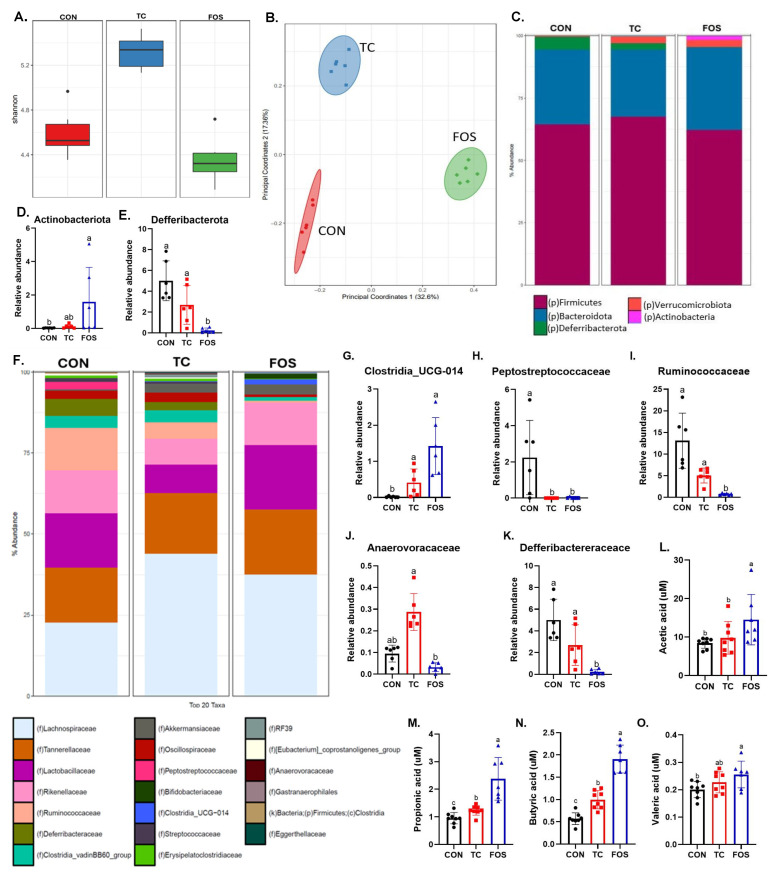
Effects of dietary supplementation with control (CON), tart cherry (TC), or fructooligosaccharides (FOS) on gut microbiota composition and short-chain fatty acids (SCFAs) concentrations. Alpha diversity assessed using the Shannon index (**A**), and beta diversity (**B**) based on Jaccard distances. Relative abundance at the phylum level (**C**), with specific differences highlighted for *Actinobacteriota* (**D**) and *Deferribacterota* (**E**). Relative abundance at the family level (**F**), and selected taxa including *Clostridia_UCG_014* (**G**), *Peptostreptococcaceae* (**H**), *Ruminococcaceae* (**I**), *Anaerovoracaceae* (**J**), and *Deferribacteraceae* (**K**). SCFA concentrations measured include acetic acid (**L**), propionic acid (**M**), butyric acid (**N**), and valeric acid (**O**). Microbiota composition was analyzed using ANCOM-BC with Benjamini–Hochberg correction for multiple comparisons. SCFA concentrations were analyzed by one-way ANOVA. Data are presented as mean ± standard error (SE). Bars not sharing the same superscript letter differ significantly (*p* < 0.05).

We next assessed how the dietary interventions altered the composition of the cecal microbiota. Simpson analysis of alpha diversity revealed that TC consumption increased (*p* < 0.01) alpha diversity compared to FOS and CON treatments (Figure 3A). FOS treatment showed a trend (*p* = 0.078) toward lower alpha diversity relative to CON (Figure 3A). Analysis of beta diversity using the Jaccard index revealed a notable separation of diet groups with PERMANOVA showing a significant (*p* < 0.01) β-diversity for each pairwise comparison (Figure 3B). The relative abundance of two bacterial phyla, Actinobacteriota and Defferibacterota, were affected by dietary treatments, while the most abundant phyla in the mouse, Firmicutes, Bacteroidota, Verrucomicrobiota, Cyanobacteria, and Proteobacteria, were not significantly altered by FOS and TC treatment (Figure 3C). The abundance of Actinobacteria phyla (*p* = 0.0084) was increased in the FOS group compared to CON, but there were no differences between FOS and TC groups (Figure 3D). Moreover, the Deferribacterota phylum was reduced (*p* < 0.01) in response to the FOS treatment compared to the CON and TC groups (Figure 3E). The relative abundance of microbial taxa at the family level was also altered by dietary treatments (Figure 3F). Diet-induced shifts in several families within the Firmicutes phylum were noted, despite no change in the overall abundance of the phylum. This included an increase in the Clostridia_UCG-014 family (*p* < 0.001) and a decrease in the Peptostreptococcaceae family (*p* < 0.0001) with the FOS and TC diets compared to the CON diet (Figure 3G,H). Similarly, within Firmicutes, Ruminococcaceae was reduced (*p* < 0.05) by the FOS diet compared to the TC and CON diets (Figure 3I), and the TC diet resulted in a higher relative abundance of Anaerovoracaceae compared to the FOS group (Figure 3J). Beyond Firmicutes, the FOS diet also reduced the relative abundance of Deferribacteraceae (*p* < 0.05) compared to the TC and CON groups (Figure 3K). The relative abundance of top family taxa such as Akkermansiaceca, Bifidobacteriaceae, Lachnospiraceae and Lactobacillaceae were not altered by either FOS or TC treatments in this study. Note that genus results are included in Supplementary Table S3.

SCFAs are produced from microbial fermentation that are known to impact gut physiology and mineral absorption [49,50], and also have positive effects on the bone [51,52]. FOS treatment resulted in a higher concentration of fecal acetic acid (*p* < 0.05) compared to the mice on the CON diet (Figure 3L). Mice on the FOS and TC dietary treatments had higher concentrations of propionic (*p* < 0.05) and butyric acid (*p* < 0.001) compared to CON, but the increase was greater with the FOS compared to the TC diet (*p* < 0.0001) (Figure 3M,N). Only the FOS diet increased (*p* < 0.05) the concentration of valeric acid in the feces compared to CON (Figure 3O).

### 3.4. FOS Enhances Treg Counts in the Ileum

Although we have previously shown that Tregs are not required for the bone protective effects of TC and FOS in young adult female mice [24], it remains important to understand how T cell populations within the intestine (i.e., ileum) and bone marrow are affected by prebiotics due to their role of immune modulation.

**Figure 4:**
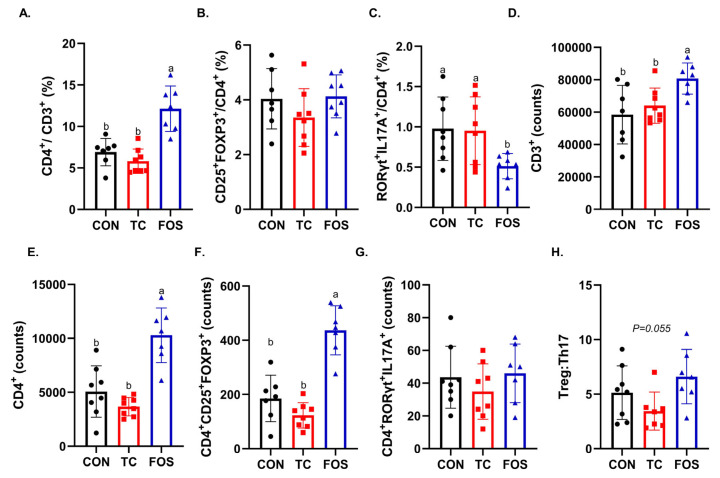
Relative abundance of T lymphocyte subsets in the lamina propria of the ileum following 10 weeks of dietary intervention with control (CON), tart cherry (TC), or fructooligosaccharides (FOS). Flow cytometry (FACS) was used to assess the percentage of (**A**) CD4^+^ T cells, (**B**) CD4^+^CD25^+^Foxp3^+^ regulatory T cells (Tregs), and (**C**) CD4^+^RORγt^+^IL-17A^+^ T helper 17 cells (Th17). Absolute counts were determined for (**D**) CD3^+^ T cells, (**E**) CD4^+^ T cells, (**F**) CD4^+^CD25^+^Foxp3^+^ Tregs, (**G**) CD4^+^RORγt^+^IL-17A^+^ Th17 cells, and (**H**) the Treg-to-Th17 cell ratio. Data are presented as mean ± standard error (SE). Bars not sharing the same superscript letter differ significantly (*p* < 0.05, one-way ANOVA with post hoc test).

Within the ileum, we found that FOS-treated mice had a greater relative abundance of CD4^+^/CD3^+^ cells compared to the TC and CON treatments (*p* < 0.0001) (Figure 4A). There was no effect of either FOS or TC on the relative abundance of CD4^+^CD25^+^FoxP3^+^, regulatory T cells (Treg) cells (Figure 4B), but FOS-treated mice exhibited a reduction in the CD4^+^RORγ^+^IL17^+^ T helper 17 cells (Th17) cells compared to other dietary treatments (Figure 4C). When T cells were expressed as absolute counts, the FOS treatment resulted in higher number (*p* < 0.05) of CD3^+^ (Figure 4D) and CD4^+^ (Figure 4E) cells compared to TC and CON treatment. FOS also increased the number of Tregs (*p* < 0.0001) compared to TC and CON (Figure 4F). There were no differences in the number of Th17 cells across the groups (Figure 4G). FOS also tended to increase the ratio of Tregs to Th17 cells compared to CON and TC (*p* = 0.055) (Figure 4H). Within the bone marrow, there were no differences in the relative abundance or absolute counts of CD4^+^CD25^+^FoxP3^+^ or CD4^+^RORγ^+^IL17^+^ T cell populations with the FOS and TC (Supplementary Table S2).

### 3.5. FOS Enhance Bone Formation and Bone Cell Density

**Figure 5:**
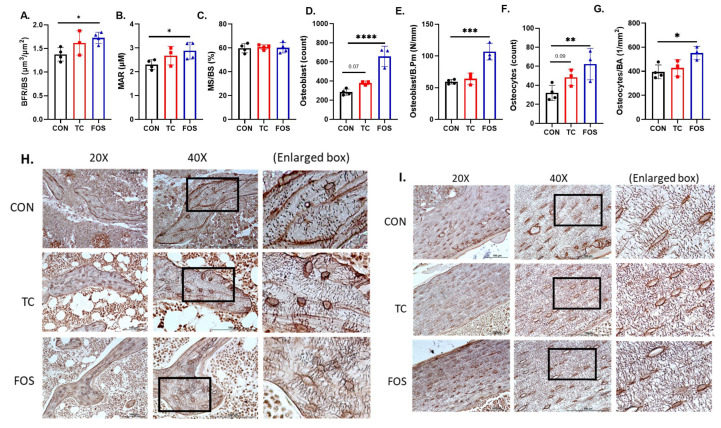
Dietary supplementation with control (CON), tart cherry (TC), or fructooligosaccharides (FOS) alters indices of dynamic bone formation, mineralization, osteoblasts, and osteocytes within the trabecular bone. (**A**) Bone formation rate per bone surface (BFR/BS), (**B**) mineral apposition rate (MAR), (**C**) mineralizing surface per bone surface (MS/BS), (**D**) osteoblast number, (**E**) osteoblast perimeter normalized to bone perimeter (Osteoblast/B.Pm), (**F**) osteocyte number, and (**G**) osteocyte density (osteocytes per bone area, BA). Representative silver nitrate staining of (**H**) trabecular and (**I**) cortical bone is also shown with the box at 40X noting the region shown in the enlarged box. Data are presented as mean ± standard error (SE). (Data was analyzed using *t*-test with bars for each significant pairwise comparison, * *p* < 0.05, ** *p* < 0.01, *** *p* < 0.001, **** *p* < 0.0001).

Although FOS improved both the trabecular and cortical bone compartments and TC enhanced trabecular bone, no significant differences due to the prebiotics were observed in serum bone biomarkers after 10 wks of treatment. This prompted us to conduct an exploratory analysis using static and dynamic bone histomorphometry on a subset of samples (n = 3–4/group) to gain some insight on the effects of these prebiotics on indices of bone formation and mineralization, as well as osteoblast and osteocyte numbers. Within the trabecular bone, the FOS diet increased the bone formation rate per bone surface (BFR/BS; *p* < 0.05) and mineral apposition rate (MAR; *p* < 0.05) compared to mice on the CON diet (Figure 5A,B).

The influence of TC on these parameters compared to CON did not reach the level of statistical significance (BFR/BS *p* = 0.10 and MAR *p* = 0.16). Neither FOS nor TC altered the mineralizing surface MS/BS (Figure 5C). Within the cortical bone, there were no effects of FOS or TC treatment on the BFR, MS/BS, or MAR on the periosteal or endocortical surfaces. To understand the influence of TC and FOS on bone, we next quantified the osteoblast and osteocyte numbers and density. Von Kossa staining revealed that FOS treatment increased the number of osteoblasts compared to the CON (Figure 5D). TC treatment resulted in a trend (*p* = 0.07) toward increasing the number of osteoblasts compared to CON, but this response did not reach the level of statistical significance (Figure 5D). FOS treatment resulted in greater osteoblasts density (*p* < 0.001) than in mice receiving the CON diet, but there were no differences between TC and CON (Figure 5E). FOS also increased the number (*p* < 0.01) (Figure 5F) and density of osteocytes (Figure 5G) within the trabecular bone compared to CON. Similar to what we observed with osteoblasts, TC tended (*p* = 0.09) to increase the osteocyte number, but not the density. Silver nitrate staining was performed to visualize the influence of dietary treatment on the number of osteocyte lacuna and the canalicular system in the trabecular (Figure 5H) and cortical (Figure 5I) bone. Representative micrographs from each treatment show that the FOS treated mice had both higher number and density of osteocyte lacunae and canalicular structures compared to TC and CON mice, with TC treated mice having more canalicular structures than CON.

### 3.6. ABX Treatment Alters Bone Phenotype and Influences Gut Production of SCFAs

To understand how the antibiotic and antifungal cocktail influences bone phenotype, we compared mice on CON diet with antibiotics (+ABX) or without antibiotics (-ABX). Mice receiving the ABX had lower whole-body BMD, BMC, and BMA (*p* < 0.01) (Table 1). Within the trabecular bone of the distal femur metaphysis and vertebra, there was no effect of ABX on BV/TV and TbTh, but there was a higher TbN (*p* < 0.01). ABX treatment reduced TbSp in the vertebrae (*p* < 0.001) and tended to have a similar effect on TbSp within the distal femur metaphysis (*p* = 0.0521), although it did not reach statistical significance. In terms of cortical bone, ABX treatment resulted in lower cortical area (*p* < 0.05), but there were no differences in cortical thickness or porosity between the two groups. The observed changes in serum bone biomarkers with ABX included a reduction in serum CTX-I, while serum P1NP levels remained unchanged. Although ABX treatment tended to reduce serum IGF-I (*p* = 0.06), there was no effect of ABX on serum IGFBP-3 or the ratio IGF-I/IGFBP-3 (Table 1). As expected, ABX treatment resulted in lower fecal concentrations of propionic (*p* < 0.01), *n*-butyric (*p* < 0.05), and *n*-valeric acid (*p* < 0.01) but had no effect on acetic acid (Table 1).

### 3.7. ABX Alters the Effect of Dietary Treatment of TC and FOS

To understand the role of the gut microbiota in mediating the response observed with TC and FOS, we first sought to confirm if the cocktail suppressed SCFA-producing bacteria and the bacterial load as indicated by gDNA. The concentrations of acetic, propionic, and butyric acids in the TC- and FOS-treated groups were reduced by ABX treatment (Table 2). ABX had a main effect on valeric acid, lowering its concentration when combined with TC and FOS. FOS treatment increased (*p* < 0.01) cecal gDNA compared to TC and CON treatments, and the suppression of this indicator of bacterial load with ABX was less in the FOS compared to the CON and TC groups (Table 2). Fecal gDNA indicated a main effect of diet (*p* < 0.01) with FOS compared to TC and CON. ABX treatment reduced (*p* < 0.0001) gDNA across all groups. In both cecal and fecal contents, FOS + ABX had higher gDNA concentration compared to other ABX-treated groups.

**Figure 6:**
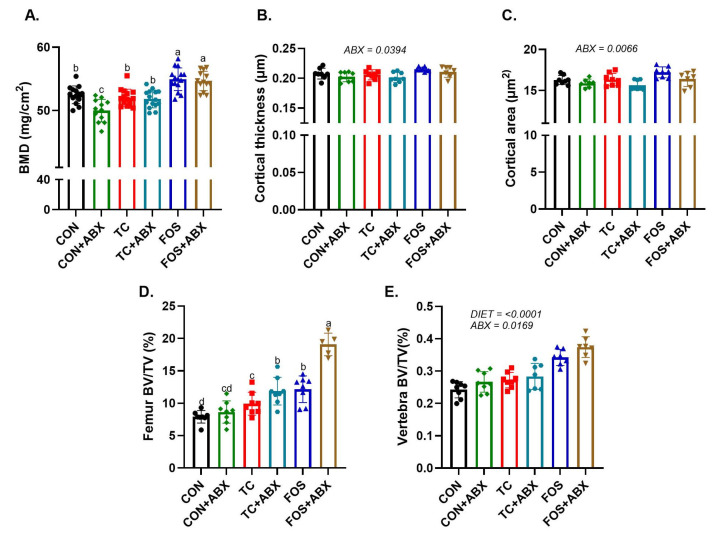
Effects of prebiotics and antibiotic (ABX) treatment on bone. Mice were fed control (CON), tart cherry (TC), or fructooligosaccharide (FOS) diets for 10 weeks, with or without ABX. Bone parameters assessed included: (**A**) whole body bone mineral density (BMD), (**B**) femur midshaft cortical thickness and (**C**) area, (**D**) femur trabecular bone volume fraction (BV/TV), and (**E**) vertebra trabecular BV/TV. Data were analyzed using two-way ANOVA. Where no significant interaction was observed, *p*-values for main effects of diet or ABX are shown. Bars are mean + SE. Bars not sharing the same superscript letter are significantly different from each other (*p* < 0.05).

Next, we evaluated the bone phenotype of mice on the TC and FOS diets with or without ABX. ABX treatment reduced whole body BMD in the CON diet group, but the TC and FOS diets prevented this loss of BMD (Figure 6A). In the mid-diaphysis of the femur, there was a main effect ABX reducing cortical thickness (Figure 6B) and cortical area in all dietary groups (Figure 6C). An unexpected interaction between ABX and diet was observed in trabecular bone in the distal femur metaphysis. Specifically, ABX combined with TC or FOS led to an increase (*p* < 0.05) in trabecular BV/TV compared to their respective non-ABX groups, with the FOS + ABX group showing the greatest increase (Figure 6D). In terms of other bone morphological parameters within the femur metaphysis, the influence of ABX on TbN followed a similar pattern, but there was no effect on TbTh (Table 3). TbSp was also reduced (*p* < 0.001) in the FOS + ABX and TC + ABX groups. In the vertebral body, there was a main effect of ABX increasing (*p* < 0.05) BV/TV (Figure 6E) and TbN (*p* < 0.001) in all dietary groups (Table 2). ABX treatment led to reduced TbSp (*p* < 0.0001) across all groups (Table 3).

## 4. Discussion

Although simple prebiotics like FOS and complex prebiotics such as tart cherry have each been shown to influence the gut–bone axis, direct comparisons of their effects are lacking, underscoring the rationale for the present study. In this study we investigated how 10 weeks of dietary supplementation with FOS or TC alters gut microbiota composition and the downstream effects on bone homeostasis in young adult mice. We also assessed the effects of these prebiotics on bone when the gut microbiota is suppressed. Our findings show that both the FOS and TC improved bone structural parameters, but the magnitude of the skeletal response was greater with FOS treatment compared to TC. FOS increased whole body BMC and BMD, which coincided with improvements in cortical thickness and cortical area. Trabecular bone within the distal femoral metaphysis and lumbar vertebral body increased with FOS by 60% and 41%, respectively. In contrast, TC consumption did not alter cortical bone in the femur or whole-body BMD or BMC; however, TC did result in a 19% higher trabecular BV/TV within the distal femur and vertebra body (12%). Previous studies have shown that both FOS and TC have osteoprotective properties, but not all studies specify whether short chain FOS was used. Compared to long-chain FOS, short-chain FOS is more rapidly fermented in the proximal colon, resulting in earlier SCFA production [53]. In this study, we used short-chain FOS, which has been shown in previous studies to have a pronounced effects on bone in similar aged female C57BL/6 mice and ovariectomized rats [24,54]. Our lab and others have reported the beneficial effects of TC on bone health in clinical and preclinical studies. Specifically, supplementing the diet with lyophilized TC powder prevented age-related bone loss in female C57BL/6 mice [27] and mitigated bone loss in a tumor necrosis factor (TNF)-overexpressing transgenic model of rheumatoid arthritis [41]. Moreover, consuming TC juice (2 x 8 fl. oz./d) for 90 d reduced biomarkers of bone resorption in postmenopausal women [40]. Although the beneficial effects of the simple prebiotic FOS and complex prebiotic TC on bone health are known, our intent with this study was to better understand the mechanisms that drive protective responses in bone.

Based on the improvements in bone density and structural parameters, we next investigated the effect of FOS and TC on bone turnover. After 10 wks of treatment, serum biomarkers of bone formation and resorption, PINP and CTX-I, respectively, were not different between treatment groups, despite the notable changes in the bone mass and structure. Previous studies with prebiotics have also shown that improvements in bone microarchitecture occur without corresponding changes in systemic biomarkers [55,56,57]. The gut microbiota has been reported to influence bone through increased serum IGF-1 [2], but we observed no differences in serum IGF-1 in this study. We did detect a reduction in serum IGFBP-3 in the FOS and TC groups compared to control, which could indicate an increase in bioavailable IGF-1. However, there were no statistically significant differences between diet groups when IGF-1 was expressed as a ratio of IGFBP-3. Due to the lack of systemic changes in bone turnover markers, we performed static and dynamic bone histomorphometry on a subset of mice to investigate local changes in the bone. Even with this small sample size, we observed that dietary FOS increased the rate of bone formation and mineralization. Similar trends were observed with TC, though these effects did not reach statistical significance. The number and density of both osteoblasts and osteocytes were higher in the FOS-fed animals. Osteoblast and osteocyte number tended to increase with TC, but not density. From a mechanistic standpoint, these results indicate the improved structural properties with FOS were driven by increased osteoblast activity that resulted in greater bone formation as well as bone mineralization rates. TC is likely having similar effects, but the magnitude of the response was not as great. Because the prebiotic components of tart cherry are embedded within a complex matrix that includes other components, it is plausible that their fermentation may be slower and occur lower within the gastrointestinal tract. Similar effects of plant matrices have been observed on fermentation kinetics in in vitro models with chicory [58]. Additionally, the increase in osteocyte density in long bones with FOS confirms our previous unanticipated finding of an increase in osteocytes within the vertebrae [24]. The implications of these findings as they relate to osteocytes and their role in mechanosensing and age-related bone loss warrant further investigation.

One of the mechanisms by which prebiotics enhance bone health is through gut microbiota modulation and the subsequent production of xenometabolites [59]. In particular, metabolites such as the SCFAs, hippuric acid, and other phenolic acids detected in circulation with prebiotic supplemented diets have been reported to have osteogenic properties in in vitro and in vivo studies [51,60,61]. To investigate how FOS and TC influence the gut microbiota, microbial populations in cecal contents were profiled. β-diversity analysis revealed there was a difference in the composition of the gut microbiota between treatments. Although the most abundant bacterial phyla in the mouse (i.e., Firmicutes, Bacteroidetes, Proteobacteria) were not altered, Actinobacteriota increased with FOS treatment and the less characterized Defferibacterota decreased by the FOS treatment. Families within the Actinobacteria phylum, most especially bifidobacteriaceae, which is commonly used as a probiotic have been positively correlated with BMD [62,63,64]. The abundance of a member of the Clostridia family (i.e., Clostridia_UCG-014), which is known to be SCFA-producing was greater with both the FOS and TC treatment. This coincided with the increased fecal SCFA concentrations (i.e., propionic and butyric acids) observed with the FOS and TC treatment. It is worth noting that fecal acetic and valeric acids were increased in the FOS treatment only. Increased SCFAs with FOS correlated with increased trabecular and cortical bone. While butyric acid has been reported to induce mesenchymal stem cells to differentiate into osteoblast and stimulate bone formation in mice [65,66], valeric acid has been recently shown to be associated with higher BMD in a population-based study [67]. Acetate, propionate, and valeric acid have been reported to promote osteoblast differentiation, although the precise mechanism through which these effects are mediated remains in question [67,68].

SCFAs influence bone by modulating the immune system, specifically increasing the Treg/Th17 ratio, which results in a more anti-inflammatory phenotype [26]. The effects of SCFAs can act through receptor-mediated pathways such as GPR109A, GPR41 and GPR43 as well as epigenetic changes (i.e., histone deacetylation) [69,70]. Interestingly, the loss of GPR109A has been shown to impair the anti-inflammatory effects of butyrate [71]. FOS and TC treatment increased bacterial load without inducing inflammation, as evidenced by the Treg and Th17 cell populations. TC polyphenols may also act through mechanisms independent of SCFAs. Following absorption and hepatic metabolism, circulating phenolic metabolites such as hippuric acid which is known to act through GPR109A has been shown to improve bone mass [60]. These SCFA-independent effects could contribute to the osteoprotective activity of TC as well. Several factors could explain why FOS produced greater skeletal effects than TC. First, the two prebiotics were matched by weight rather than by FOS content, which means the amount of FOS in the TC diet was lower than in the FOS diet. Second, the structural simplicity of FOS allows for rapid fermentation and earlier SCFA release, whereas the prebiotic compounds in TC are embedded within a complex food matrix that may slow fermentation and shift metabolism further along the gut. Third, these differences in substrate composition likely influenced the gut microbial community, leading to distinct SCFA profiles and potentially other metabolites that contribute to divergent skeletal responses. These findings support the idea that dietary interventions with FOS and TC enhance bone health by modulating the gut microbiota to favor the production of metabolites that have positive effects on osteoblasts.

To determine whether alterations in the gut microbiota and SCFA production are required to mediate the response observed with FOS and TC, we utilized a broad-spectrum antibiotic cocktail known to suppress microbiota in combination with an antifungal to prevent fungal overgrowth [43,44,45]. In mice consuming the control diet, the cocktail reduced cortical bone area but had no effect on trabecular BV/TV in either the distal femur metaphysis or vertebral body. As expected, the addition of the ABX cocktail in mice fed FOS or TC diets resulted in reduced cortical thickness and area. However, in the trabecular bone compartment of the distal femur, an unexpected increase in trabecular BV/TV was observed when the cocktail was combined with the TC or FOS diets. This increase in trabecular bone occurred even though the cocktail reduced fecal SCFA concentrations across all groups, which is consistent with the suppression of SCFA-producing taxa. The trabecular bone response in the femur to the antibiotic/antifungal cocktail suggests that the bone effects at this site are either not mediated by SCFAs alone or that the combination of a prebiotic with this cocktail results in unintended effects on the gut (e.g., altering gut permeability, nutrient absorption or gut-derived metabolites) which acted on the trabecular bone. The cocktail used in this study has been shown to reduce both Gram-negative and -positive bacteria, including the SCFA producing lactobacillus [72]. The cecal gDNA levels indicated the bacterial load was suppressed by 90% and 95% in the CON and TC groups treated with the cocktail, respectively, but with the FOS treated mice only 80% suppression was achieved. Unfortunately, characterization of the gut microbiota for the mice receiving the antibiotics was not performed. The effect of the cocktail on trabecular bone was only observed in the FOS and TC groups, raising the question of whether these diets enhance the absorption or bioavailability of some component of the cocktail or nutrients that directly affect bone cellular activity. Many studies utilizing antibiotics to ablate the microbiota have not incorporated antifungal agents to suppress the overgrowth of fungi that can occur when using broad spectrum antibiotics. Although some antifungal agents exhibit histone deacetylase inhibition (HDACi) and HDACi can promote osteoblast differentiation and bone formation [66,73], we are not aware of reports that amphotericin B acts via this mechanism. It is possible that the antifungal and antibiotics have contributed to the increased trabecular bone independent of microbial suppression. Although the use of broad-spectrum antibiotics to suppress the gut microbiota is a common strategy for studying microbiota-dependent effects, our study points to the fact that this model has notable limitations when combined with prebiotics and may have unintended systemic effects, including altered nutrient metabolism and immune responses [74,75]. Additionally, this approach does not fully deplete gut microbes and may result in partial suppression or alterations in the microbial communities. As such, the influence of TC and FOS on bone health in the antibiotic cocktails-treated groups should be interpreted with caution. While germ-free models offer the advantage of complete microbiota elimination, they also present limitations such as altered immune and metabolic responses, which complicate interpretation of diet, microbiota, and bone interactions. In contrast, the ABX model used here avoids some of these confounders but introduces other uncertainties, including potential off-target effects on nutrient absorption and gut barrier function that complicates interpretation.

## 5. Conclusions

Our findings demonstrate that the beneficial effects of FOS and TC on bone are, in part, mediated by increased bone formation by osteoblasts and bone mineralization. Both prebiotics altered the gut microbiota and increased fecal SCFAs, suggesting a microbiota-associated mechanism. However, because the antibiotic suppression model has the potential to broadly perturb host physiology in addition to depleting the microbiota, it limits our ability to draw causal inferences and leaves the question of whether or not the observed skeletal response to the ABX is microbiota dependent. Future studies using germ-free or fecal transplantation models will be important to more definitively establish the microbiota’s role.

Notably, we observed an increase in osteocyte density with FOS, which may be important given the critical role of osteocytes in maintaining bone quality and orchestrating bone remodeling. Enhancing osteocyte populations during youth, as demonstrated in young adult mice in this study, could provide a strategy to strengthen the skeletal foundation early in life and potentially mitigate bone loss with age. Overall, this study indicates that FOS and TC are dietary interventions that warrant further investigation for their potential to support bone health across the lifespan.

## Figures and Tables

**Table 1:** ABX Effects on Bone and SCFA Profiles in Mice on Control Diet.

	No ABX	ABX	*p*-Value
Bone Densitometry			
BMD (mg/cm^2^)	52.48 ± 0.37	50.00 ± 0.51	0.0001
BMC (mg)	48.41 ± 0.82	44.67 ± 1.00	0.0043
BMA (cm^2^)	9.22 ± 0.12	8.54 ± 0.15	0.0012
Vertebra Trabecular Bone			
BV/TV (%)	24.28 ± 0.89	26.67 ± 1.12	0.1193
TbTh (µm)	0.058 ± 0.0009	0.056 ± 0.001	0.2304
TbN (1/mm^2^)	4.32 ± 0.12	4.87 ± 0.12	0.0013
TbSp (µm)	0.23 ± 0.007	0.19 ± 0.006	0.0009
Femur Trabecular Bone			
BV/TV (%)	7.9 ± 0.34	8.64 ± 0.61	0.3545
TbTh (µm)	0.049 ± 0.001	0.048 ± 0.001	0.4017
TbN (1/mm^2^)	3.36 ± 0.04	3.55 ± 0.06	0.0498
TbSp (µm)	0.29 ± 0.004	0.27 ± 0.004	0.0521
Femur Cortical Bone			
Cortical thickness (µm)	0.21 ± 0.003	0.20 ±0.003	0.1349
Cortical area (µm^2^)	16.28 ± 0.19	15.03 ± 0.86	0.0343
Cortical porosity (%)	3.5 ± 0.06	3.6 ± 0.04	0.4486
Serum Bone Biomarkers			
CTX-I (ng/mL)	14.51 ± 0.58	11.89 ± 0.78	0.0410
P1NP (ng/mL)	16.07 ± 1.39	15.25 ± 1.94	0.7427
IGF-I (pg/mL)	386,870 ± 24,488	307,533 ± 40,932	0.0629
IGFBP-3 (pg/mL)	280,663 ± 20,424	275,261 ± 30,444	0.9155
IGF-I/IGFBP-3 (pg/mL)	1.42 ± 0.09	1.03 ± 0.10	0.2558
Fecal SCFAs (µmol/g)			
Acetic acid	8.43 ± 0.46	6.84 ± 0.97	0.1047
Propionic acid	0.95 ± 0.07	0.69 ± 0.05	0.0016
Butyric acid	0.57 ± 0.05	0.38 ± 0.03	0.0399
Valeric acid	0.20 ± 0.01	0.12 ± 0.03	0.0065

Data presented as mean ± SE. n = 8/group. Abbreviations: No ABX = Control diet without antibiotics in drinking water; ABX = Control diet with antibiotics in drinking water. (*p* < 0.05, *t*-test).

**Table 2:** Fecal SCFA and gDNA Concentrations.

	CON		TC		FOS		*p*-Values		
	No ABX	ABX	No ABX	ABX	No ABX	ABX	DIET	ABX	Diet × ABX
Fecal SCFAs (µmol/g)									
Acetic acid	8.4 ± 0.5 ^b^	6.9 ± 1.0 ^bc^	8.6 ± 1.1 ^b^	3.6 ± 0.2 ^cd^	14.5 ± 2. 5 ^a^	3.1 ± 0.2 ^d^	0.0095	<0.0001	<0.0001
Propionic acid	1.0 ± 0.17 ^bc^	0.7 ± 0.1 ^cd^	1.2 ± 0.1 ^b^	0.5 ± 0.1 ^d^	2.2 ± 0.3 ^a^	0.6 ± 0.1 ^d^	0.0002	<0.0001	0.0002
Butyric acid	0.6 ± 0.05 ^c^	0.4 ± 0.0 ^d^	1.0 ± 0.1 ^b^	0.3 ± 0.1 ^d^	1.9 ± 0.1 ^a^	0.3 ± 0.0 ^d^	0.0004	<0.0001	<0.0001
Valeric acid	0.2 ± 0.0	0.1 ± 0.0	0.2 ± 0.0	0.1 ± 0.0	0.3 ± 0.0	0.1 ± 0.0	0.7281	<0.0001	0.0786
gDNA Conc (ng/μL)									
Cecal	142.7 ± 34.1 ^b^	13.5 ± 2.5 ^d^	150.1 ± 40.6 ^b^	7.8 ± 0.7 ^d^	237.0 ± 36.1 ^a^	48.4 ± 9.2 ^c^	0.0001	<0.0001	0.0276
Fecal	144.1 ± 8.7	51.0 ± 2.3	174.4 ± 24.5	55.8 ± 21.7	191.3 ± 10.4	146.8 ± 18.0	0.0013	<0.0001	0.1031

Data are presented as mean ± standard error (SE). Abbreviations: CON, control diet; TC, tart cherry diet; FOS, fructooligosaccharide diet; ABX, antibiotics. Statistical significance was defined as *p* < 0.05 for main effects of diet or ABX, and for diet × ABX interactions. The superscript letters differ significantly.

**Table 3:** Effects of Diet and Antibiotic Treatment on Femoral and Lumbar (L5) Vertebral Bone Microarchitecture.

	CON		TC		FOS		*p*-Values		
	No ABX	ABX	No ABX	ABX	No ABX	ABX	DIET	ABX	Diet × ABX
L5 Trabecular Bone									
BV/TV (%)	24.29 ± 0.90	26.68 ± 1.12	27.24 ± 0.84	28.36 ± 1.53	34.23 ± 0.93	37.43 ± 1.21	<0.0001	0.0169	0.6458
TbN (1/mm^2^)	4.32 ± 0.12	4.88 ± 0.12	4.56 ± 0.13	4.90 ± 0.07	4.64 ± 0.13	4.91 ± 0.11	0.3074	0.0002	0.4321
TbTh (mm)	0.06 ± 0.00 ^c^	0.06 ± 0.00 ^c^	0.06 ± 0.00^c^	0.06 ± 0.00 ^c^	0.07 ± 0.00 ^b^	0.07 ± 0.00 ^a^	<0.0001	0.5109	0.0035
TbSp (mm)	0.23 ± 0.01	0.20 ± 0.01	0.22 ± 0.01	0.20 ± 0.01	0.21 ± 0.01	0.19 ± 0.01	0.0765	<0.0001	0.7162
Femur Trabecular Bone									
BV/TV (%)	7.90 ± 0.34 ^d^	8.64 ± 0.61 ^cd^	9.93 ± 0.62 ^c^	11.87 ± 0.75 ^b^	12.16 ± 0.72 ^b^	19.09 ± 0.78 ^a^	<0.0001	<0.0001	0.0150
TbN (1/mm^2^)	3.37 ± 0.05 ^d^	3.55 ± 0.07 ^cd^	3.63 ± 0.04 ^c^	4.00 ± 0.09 ^b^	3.98 ± 0.09 ^b^	4.65 ± 0.10 ^a^	<0.0001	<0.0001	0.0316
TbTh (mm)	0.05 ± 0.00	0.05 ± 0.00	0.05 ± 0.00	0.05 ± 0.00	0.05 ± 0.00	0.05 ± 0.00	0.0009	0.5483	0.7425
TbSp (mm)	0.29 ± 0.00 ^c^	0.28 ± 0.01 ^ab^	0.27 ± 0.00 ^b^	0.24 ± 0.01 ^c^	0.24 ± 0.01 ^c^	0.19 ± 0.01 ^d^	<0.0001	<0.0001	0.0091

Data are presented as mean ± standard error (SE). Abbreviations: CON, control diet; TC, tart cherry diet; FOS, fructooligosaccharide diet; ABX, antibiotics. Statistical significance was defined as *p* < 0.05 for main effects of diet or ABX, and for diet × ABX interactions. The superscript letters differ significantly.

## Data Availability

Data associated with this manuscript will be made available upon request.

## Supplementary Materials

The following supporting information can be downloaded at: https://www.mdpi.com/article/10.3390/nu17172829/s1, Table S1: Additional Body Composition and Bone Structural Characteristics. Table S2: T-Lymphocyte Absolute Counts and Percentage in Bone Marrow. Table S3: Effects of Prebiotics on the Relative Abundance at the Genus Level.
