# Supplementary material for: Dietary Tart Cherry and Fructooligosaccharides Promote Bone Health via the Gut Microbiota and Increased Bone Formation
Source: Nutrients. 2025 Aug 30;17(17):2829. doi: 10.3390/nu17172829 (PMC12430659; doi:10.3390/nu17172829)
Supplement: Supplementary file 1 [file nutrients-17-02829-s001.zip › nutrients-3825362-supplementary.pdf]

Supplementary Table S1: Additional Body Composition and Bone Structural Characteristics

	CON	TC	FOS	P-value
**Body Composition**				
Percent Fat (%)	38.81 ± 1.52[a]	42.70 ± 1.43[a]	25.60 ± 1.00[b]	<0.0001
**L5 Trabecular Bone**				
TbTh (μm)	0.058 ± 0.0009[b]	0.059 ± 0.0006[b]	0.066 ± 0.0008[a]	<0.0001
TbN (1/mm^2)	4.33 ± 0.12	4.56 ± 0.13	4.64 ± 0.13	0.2102
TbSp (μm)	0.23 ± 0.007	0.22 ± 0.007	0.21 ± 0.007	0.2144
ConnDenn (1/mm^3)	151.35 ± 9.44[b]	181.63 ± 9.45[a]	176.18 ± 6.12[a]	0.0426
SMI	0.85 ± 0.075[a]	0.58 ± 0.080[b]	-0.15 ± 0.12[c]	<0.0001
**Femur Trabecular Bone**				
TbTh (μm)	0.050 ± 0.0006[a]	0.048 ± 0.0007[b]	0.052 ± 0.0007[a]	0.0023
TbN (1/mm^2)	3.37 ± 0.045[c]	3.63 ± 0.039[b]	4.03 ± 0.080[a]	<0.0001
TbSp (μm)	0.29 ± 0.004[a]	0.27 ± 0.004[b]	0.24 ± 0.005[c]	<0.0001
ConnDenn (1/mm^3)	60.18 ± 3.32[c]	89.12 ± 6.69[b]	124.40 ± 6.74[a]	<0.0001
SMI	2.55 ± 0.070[a]	2.31 ± 0.073[ab]	2.22 ± 0.11[b]	0.0339
**Femur Cortical Bone**				
Cortical Porosity	3.51 ± 0.062[ab]	3.66 ± 0.048[a]	3.40 ± 0.062[b]	0.0151

Supplementary Table S2: T-Lymphocyte Absolute Counts and Percentage in Bone Marrow

	CON	TC	FOS	P-value
**Bone Marrow T-Lymphocytes**				
$CD3^+$ (count)	38068.25 ± 2051.92	33450.14 ± 3308.22	29486.00 ± 3676.10	0.1522
$CD4^+$ (count)	7466 ± 676.06	6187.75 ± 555.19	5491.83 ± 579.61	0.0816
$CD4^+CD25^+FOXP3^+$ (count)	3052.14 ± 92.18	2455.88 ± 224.72	2833.86 ± 432.15	0.0779
$CD4^+RORgt^+IL17A^+$ (count)	112.71 ± 26.00	143.00 ± 18.89	93.43 ± 24.34	0.1712
Treg: Th17	34.56 ± 5.90	18.53 ± 2.36	40.08 ± 7.95	0.0424
$CD4^+/CD3^+$ (%)	19.45 ± 0.83	19.97 ± 1.33	21.85 ± 1.14	0.2791
$FOXP3^+/CD4^+$ (%)	45.34 ± 1.62	42.16 ± 0.72	45.09 ± 1.09	0.1695
$RORgt^+IL17A^+/CD4^+$ (%)	1.74 ± 0.31	2.39 ± 0.30	1.65 ± 0.34	0.2106

Supplementary Table S3: Effects of Prebiotics on the Relative Abundance at the Genus Level

Genus	CON	TC	FOS	P-value CON vs. TC	P-value CON vs. FOS	P-value TC vs. FOS
Eubacterium_coprostanoligenes_group	0.61 ± 0.39	0.58 ± 0.17	0.04 ± 0.02	NS	NS	NS
Akkermansia	0.46 ± 0.19	2.64 ± 1.49	2.84 ± 0.89	NS	NS	NS
Eubacterium_brachy_group	0.07 ± 0.01	0.04 ± 0.01	0.03 ± 0.01	NS	NS	NS
Family_XIII_UCG-001	0.01 ± 0.01	0.2 ± 0.03	0 ± 0	<0.0001	1	<0.0001
Bifidobacterium	0 ± 0	0 ± 0	1.52 ± 0.83	NS	NS	NS
Clostridia_UCG-014	0.01 ± 0.01	0.41 ± 0.15	1.42 ± 0.32	<0.0001	<0.0001	0.21607
Clostridia_vadinBB60_group	3.93 ± 0.98	3.67 ± 0.99	1.03 ±0.32	NS	NS	NS
Mucispirillum	5 ± 0.78	2.69 ± 0.76	0.23 ± 0.09	0.9223	<0.0001	0.00305
Adlercreutzia	0.02 ± 0.01	0.07 ± 0.03	0.07 ± 0.01	NS	NS	NS
Enterococcus	0.1 ± 0.04	0.02 ± 0.01	0.02 ± 0.01	NS	NS	NS
Erysipelatoclostridium	0.74 ± 0.18	0.73 ± 0.15	0.22 ± 0.06	NS	NS	NS
Gastranaerophilales	0.03 ± 0.01	0.2 ± 0.07	0.07 ± 0.03	NS	NS	NS
A2	0.01 ± 0.01	0.2 ± 0.07	0.24 ± 0.07	0.0164	0.00028	1
Acetatifactor	0.91 ± 0.42	0.87 ± 0.55	0 ± 0	NS	NS	NS
ASF356	0 ± 0	0.52 ± 0.16	0.28 ± 0.16	<0.0001	0.00427	1
Blautia	5.58 ± 1.03	4.45 ± 0.71	0.79 ± 0.23	1	0.00113	0.04051
Eubacterium_xylanophilum_group	0.14 ± 0.03	0.45 ± 0.22	0.01 ± 0.01	NS	NS	NS
GCA_900066575	0.71 ± 0.16	0.68 ± 0.09	0.85 ± 0.23	NS	NS	NS
Lachnoclostridium	1.69 ± 0.25	1.92 ± 0.3	2.92 ± 0.99	NS	NS	NS
Lachnospiraceae_PC50020_group	0.02 ± 0.01	0.1 ± 0.01	0.03 ± 0.01	0.5425	0.0230	1
Lachnospiraceae_NK4A136_group	1.77 ± 0.53	11.01 ± 1.32	1.32 ± 0.43	0.0025	1	0.00543
Lachnospiraceae_UCG-006	0.05 ± 0.02	0.52 ± 0.14	0.14 ± 0.06	NS	NS	NS
Marvinbryantia	0 ± 0	0.02 ± 0.01	0.17 ± 0.06	1	<0.0001	0.01282
Roseburia	0.37 ± 0.1	0.87 ± 0.23	0.69 ± 0.33	NS	NS	NS
Tuzzerella	0.16 ± 0.03	0 ± 0	0.02 ± 0.01	<0.0001	0.00021	0.43225
Uncultured	1.03 ± 0.12	0.7 ± 0.1	0.25 ± 0.03	0.0024	0.01761	1
Lactobacillus	16.84 ± 6.37	8.76 ± 3.21	19.84 ± 3.72	NS	NS	NS
Colidextribacter	0.72 ± 0.14	0.95 ± 0.17	0.09 ± 0.03	1	0.0002	0.00077
Intestinimonas	0.1 ± 0.02	0.16 ± 0.06	0.01 ± 0.01	1	0.00121	0.00579

Oscillibacter	0.28 ± 0.01	0.12 ± 0.03	0.42 ± 0.15	NS	NS	NS
Uncultured	0.86 ± 0.15	0.54 ± 0.24	0.17 ± 0.06	1	0.00794	0.61185
Uncultured	0.03 ± 0	0.03 ± 0.01	0.07 ± 0.01	NS	NS	NS
Romboutsia	2.24 ± 0.84	0 ± 0	0.02 ± 0.02	<0.0001	<0.0001	1
RF39	0.01 ± 0.01	0.67 ± 0.26	0.09 ± 0.02	<0.0001	0.0013	0.20183
Alistipes	13.19 ± 1.45	8.18 ± 2.07	13.09 ± 1.22	NS	NS	NS
Uncultured	9.2 ± 2.86	3.09 ± 0.76	0.41 ± 0.07	NS	NS	NS
Anaerotruncus	1.81 ± 0.3	0.56 ± 0.14	0.03 ± 0.01	0.1616	<0.0001	0.00013
Incertae_Sedis	0.33 ± 0.05	0.54 ± 0.08	0.12 ± 0.03	NS	NS	NS
Uncultured	1.65 ± 0.21	0.79 ± 0.2	0.17 ± 0.05	0.5147	<0.0001	0.17154
Lactococcus	1.13 ± 0.26	0.58 ± 0.14	0.2 ± 0.03	NS	NS	NS
Parabacteroides	16.94 ± 0.02	18.66 ± 1.02	20.04 ± 2.68	NS	NS	NS
UCG-010	0.09 ± 0.02	0.1 ± 0.01	0.08 ± 0.03	NS	NS	NS
